# A Case of Massive Retroperitoneal Hematoma After High-Energy Trauma

**DOI:** 10.7759/cureus.51080

**Published:** 2023-12-25

**Authors:** Tomohiro Nakajima, Keitaro Nakanishi, Keisuke Harada, Eichi Narimatsu, Nobuyoshi Kawaharada

**Affiliations:** 1 Cardiovascular Surgery Department, Sapporo Medical University, Sapporo, JPN; 2 Emergency Department, Sapporo Medical University, Sapporo, JPN

**Keywords:** traffic accident, parabdominal rectus muscle approach, peritoneal, trauma, hematoma

## Abstract

A 66-year-old female suffered from high-energy trauma due to a traffic accident, resulting in injuries to the iliac artery and the superior mesenteric artery. She underwent endovascular embolization for vascular occlusion and an open surgical procedure to control bleeding from the superior mesenteric artery. A substantial retroperitoneal hematoma was observed on the right side, making primary closure challenging. A hematoma evacuation procedure was performed using a right retroperitoneal approach, successfully relieving the compression from the posterior aspect.

## Introduction

In patients with massive retroperitoneal hematoma, the retroperitoneum can be reached by a parabdominal rectus muscle approach. Abdominal midline incision is a conventional approach, offering the advantage of not requiring additional incisions. However, in cases where intestinal damage is suspected, there is a risk of communication between the abdominal cavity and the right retroperitoneum. On the other hand, the right-side abdominal approach has the advantage of avoiding communication between the retroperitoneal space and the abdominal cavity, but it comes with the drawback of requiring an additional incision in the skin. Unlike when the retroperitoneum is reached via the abdominal cavity, this approach can remove the hematoma without direct contact with the intestine.

## Case presentation

**Figure 1:**
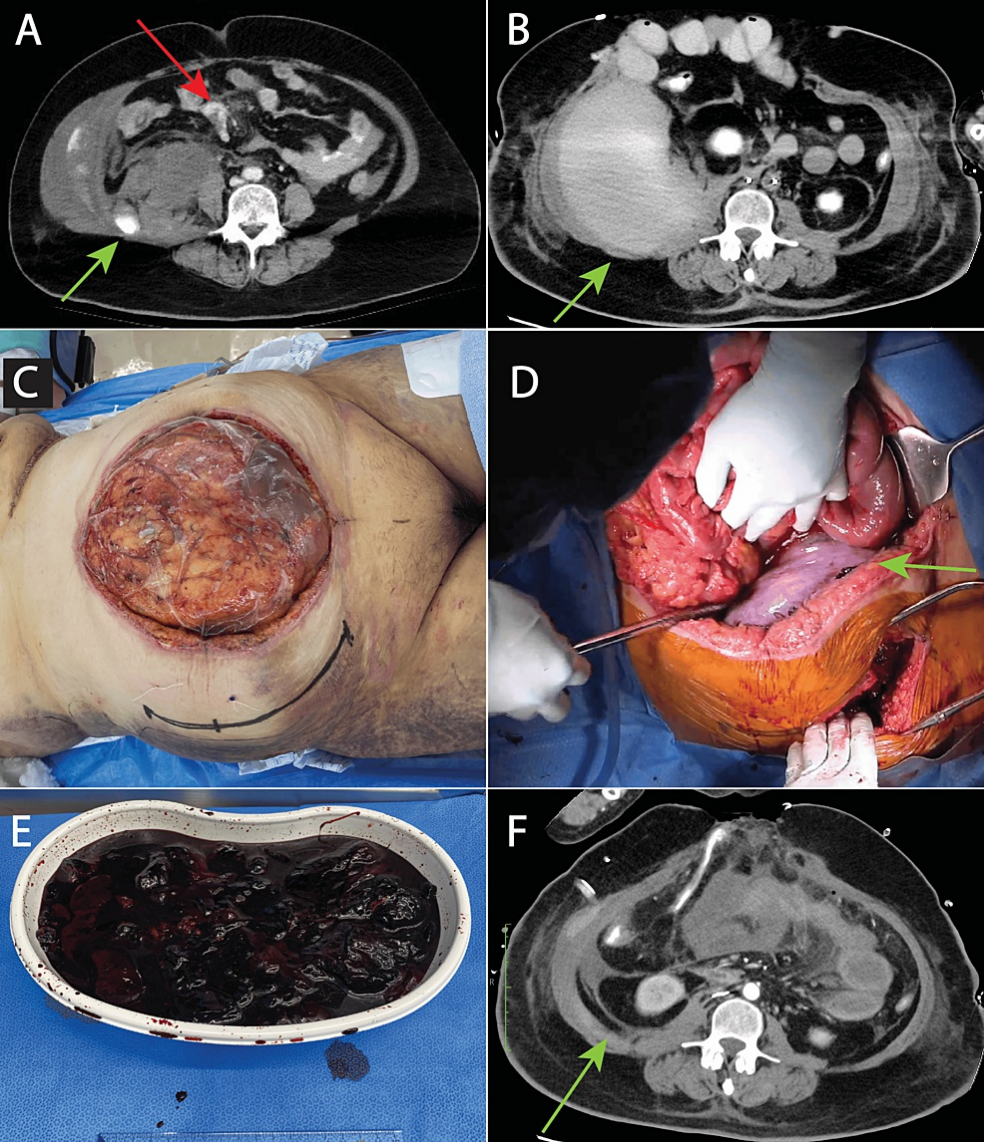
Perioperative findings (A) Preoperative computed tomography scan obtained at a nearby hospital after trauma showing hemorrhage from a damaged mesenteric artery (red arrow) and retroperitoneal hemorrhage (green arrow). (B) A post-laparotomy computed tomography scan showing a massive hematoma in the right retroperitoneum (green arrow). (C) Preoperative appearance of the patient before retroperitoneal surgery; the abdomen is open with the location of the parabdominal rectus muscle marked. The patient's cephalic side is on the left and her caudal side is on the right. (D) The left retroperitoneum was opened intraoperatively, and intraperitoneal observation revealed a bulging right retroperitoneum. (E) A dark red hematoma weighing 1200 g was manually removed. (F) Postoperative computed tomography scan showing marked reduction of the hematoma in the right retroperitoneum.

A 66-year-old female driver sustained multiple traumatic injuries (pelvic bone fracture, rib fracture, and radius bone fracture) in a road traffic accident. She was brought to our emergency department with a mesenteric artery injury and pelvic hemorrhage by enhanced computed tomography (Figure 1A). We decided to perform endovascular embolization for the pelvic hemorrhage and open hemostasis for the injury to the mesenteric artery in the operating room. We treated her with emergency physicians, orthopedic surgeons, and radiology.

Cardiac arrest occurred when endovascular treatment was started, and cardiopulmonary resuscitation was performed. The heartbeat resumed after 26 minutes, and embolization of the damaged lumbar artery and hemostasis of the mesenteric artery was performed via an open abdominal approach. The abdominal wall could not be closed, and the patient was managed with an open abdomen and a continuous negative pressure device [1]. Postoperative computed tomography showed a large hematoma in the right retroperitoneum that was compressing the abdominal cavity (Figure 1B). We anticipated that the patient would require management with an open abdomen for several weeks if we waited for the hematoma to be resorbed spontaneously. We considered approaching the right retroperitoneum through the abdominal cavity but thought that there was a risk of infection because the abdominal cavity and retroperitoneum were in contact with each other. Therefore, in consultation with the patient, we made a plan that entailed making an incision in the right parabdominal rectus muscle separately from the midline of the abdomen to reach the right retroperitoneum and remove the retroperitoneal hematoma by intraperitoneal observation. A dark red hematoma was observed on the right retroperitoneum. It was soft and could be easily removed manually. Six days after the accident, we performed this surgery simultaneously with the cardiovascular surgery and emergency department in the operation room.

A 15-cm incision was made through the right para-rectus muscle, and the right retroperitoneum was reached (Figure 1C). The hematoma was removed after checking its status from the abdominal cavity (Figure 1D). A dark red hematoma was manually evacuated, and the volume removed was 1200 g. After placing a 19-Fr drain, the retroperitoneum was closed and the operation was completed. Although most of the retroperitoneal hematoma was removed, there was swelling of the intestinal tract and the abdominal wall could not be closed at that time. Furthermore, there was necrotic degeneration of part of the intestinal tract, which was resected and a colostomy was constructed. Computed tomography on the day after surgery showed that most of the right retroperitoneal hematoma had been removed (Figure 1F). Multidisciplinary management was continued thereafter, and the abdominal wall was closed 18 days after the injury. The patient was in a state of prolonged unconsciousness and was unresponsive. Postoperatively, the patient experienced pneumonia and delayed wound healing at the surgical site, necessitating a prolonged period before transfer to another medical facility. On postoperative day 81, she was transferred to a rehabilitation hospital.

## Discussion

High-energy traumatic injuries have a poor prognosis [2]. This type of poly-trauma is likely to cause serious and sometimes fatal injuries, including fractures, brain injury, internal organ damage, spinal cord injury, major hemorrhage, and organ rupture. Treatment includes surgery, blood transfusions, emergency first aid, and rehabilitation, and a multidisciplinary team approach is critical.

Early and appropriate medical intervention is necessary [3]. It is widely recognized that interventional radiology is useful in trauma care, especially when performing transcatheter arterial embolization [4] for control of bleeding induced by blunt trauma injuries to abdominal parenchymal organs and pelvic injuries [5].

The retroperitoneal approach through a parabdominal rectus muscle incision is a routine approach for cardiovascular surgeons and is sometimes used in thoracoabdominal aortic surgery and abdominal aortic and iliac artery surgery [6].

In this case, the abdominal cavity was under compression due to a massive retroperitoneal hematoma. Anticipating a protracted recovery period until the hematoma was absorbed before closure, a decision was made to expedite closure by removing the hematoma through a right retroperitoneal approach. Although closure was achieved on day 18, it cannot be definitively concluded that it was performed earlier than if waiting for natural absorption [7]. However, it is speculated that the course may have been faster than waiting for spontaneous resolution.

For cases involving a substantial retroperitoneal hematoma, as in this instance, the approach of removing the hematoma through the retroperitoneum is considered a reasonable method that does not adversely affect the abdominal cavity.

## Conclusions

We encountered a case involving a 66-year-old female who suffered from a right retroperitoneal hematoma due to high-energy trauma. A surgical procedure for hematoma evacuation was performed using a right retroperitoneal approach. A total of 1200 grams of hematoma was successfully removed from the retroperitoneal space, allowing for increased intra-abdominal space. In cases of massive retroperitoneal hematoma, this approach provided a means to separate the surgical field more effectively compared to a midline laparotomy with a large peritoneal incision.
